# Latent Heterogeneity of Online Sexual Experiences and Associations With Sexual Risk Behaviors and Behavioral Health Outcomes in Chinese Young Adults: Cross-Sectional Study

**DOI:** 10.2196/50020

**Published:** 2024-01-26

**Authors:** Ted C T Fong, Derek Yee Tak Cheung, Edmond Pui Hang Choi, Daniel Y T Fong, Rainbow T H Ho, Patrick Ip, Man Chun Kung, Mona Wai Cheung Lam, Antoinette Marie Lee, William Chi Wai Wong, Tai Hing Lam, Paul S F Yip

**Affiliations:** 1 Centre on Behavioral Health Faculty of Social Sciences The University of Hong Kong Hong Kong China (Hong Kong); 2 School of Nursing Li Ka Shing Faculty of Medicine The University of Hong Kong Hong Kong China (Hong Kong); 3 Department of Social Work & Social Administration Faculty of Social Sciences The University of Hong Kong Hong Kong China (Hong Kong); 4 Department of Paediatrics & Adolescent Medicine Li Ka Shing Faculty of Medicine The University of Hong Kong Hong Kong China (Hong Kong); 5 Family Planning Association of Hong Kong Hong Kong China (Hong Kong); 6 Department of Psychology Faculty of Social Sciences The University of Hong Kong Hong Kong China (Hong Kong); 7 Department of Family Medicine & Primary Care Li Ka Shing Faculty of Medicine The University of Hong Kong Hong Kong China (Hong Kong); 8 School of Public Health Li Ka Shing Faculty of Medicine The University of Hong Kong Hong Kong China (Hong Kong); 9 Centre for Suicide Research and Prevention Faculty of Social Sciences The University of Hong Kong Hong Kong China (Hong Kong)

**Keywords:** Hong Kong, latent class analysis, mediation, mental health, sex knowledge, sexual risk behaviors, sexually transmitted infections, structural equation modeling, youth sexuality

## Abstract

**Background:**

Online sexual experiences (OSEs) are becoming increasingly common in young adults, but existing papers have reported only on specific types of OSEs and have not shown the heterogeneous nature of the repertoire of OSEs. The use patterns of OSEs remain unclear, and the relationships of OSEs with sexual risk behaviors and behavioral health outcomes have not been evaluated.

**Objective:**

This study aimed to examine the latent heterogeneity of OSEs in young adults and the associations with sexual risk behaviors and behavioral health outcomes.

**Methods:**

The 2021 Youth Sexuality Study of the Hong Kong Family Planning Association phone interviewed a random sample of 1205 young adults in Hong Kong in 2022 (male sex: 613/1205, 50.9%; mean age 23.0 years, SD 2.86 years) on lifetime OSEs, demographic and family characteristics, Patient Health Questionnaire-4 (PHQ-4) scores, sex-related factors (sexual orientation, sex knowledge, and sexual risk behaviors), and behavioral health outcomes (sexually transmitted infections [STIs], drug use, and suicidal ideation) in the past year. Sample heterogeneity of OSEs was analyzed via latent class analysis with substantive checking of the class profiles. Structural equation modeling was used to examine the direct and indirect associations between the OSE class and behavioral health outcomes via sexual risk behaviors and PHQ-4 scores.

**Results:**

The data supported 3 latent classes of OSEs with measurement invariance by sex. In this study, 33.1% (398/1205), 56.0% (675/1205), and 10.9% (132/1205) of the sample were in the abstinent class (minimal OSEs), normative class (occasional OSEs), and active class (substantive OSEs), respectively. Male participants showed a lower prevalence of the abstinent class (131/613, 21.4% versus 263/592, 44.4%) and a higher prevalence of the active class (104/613, 17.0% versus 28/592, 4.7%) than female participants. The normative class showed significantly higher sex knowledge than the other 2 classes. The active class was associated with male sex, nonheterosexual status, higher sex desire and PHQ-4 scores, and more sexual risk behaviors than the other 2 classes. Compared with the nonactive (abstinent and normative) classes, the active class was indirectly associated with higher rates of STIs (absolute difference in percentage points [Δ]=4.8%; *P*=.03) and drug use (Δ=7.6%; *P*=.001) via sexual risk behaviors, and with higher rates of suicidal ideation (Δ=2.5%; *P*=.007) via PHQ-4 scores.

**Conclusions:**

This study provided the first results on the 3 (abstinent, normative, and active) latent classes of OSEs with distinct profiles in OSEs, demographic and family characteristics, PHQ-4 scores, sex-related factors, and behavioral health outcomes. The active class showed indirect associations with higher rates of STIs and drug use via sexual risk behaviors and higher rates of suicidal ideation via PHQ-4 scores than the other 2 classes. These results have implications for the formulation and evaluation of targeted interventions to help young adults.

## Introduction

Digital technology has transformed the communication styles and lives of young generations who have grown up with the internet. During emerging adulthood, individuals experience intense developments in the physical, cognitive, and psychosocial domains [1], which include greater interactions with the social environment, self-identity development, and sexuality exploration [2]. Emerging adults typically establish their sexual identity and patterns of health and risk behaviors, which have health implications for later life. The internet and social media are essential for forming social relationships among these adults.

Popular culture in Western countries has embraced uncommitted sex, such as casual sexual relationships and experiences, among young adults [3]. Online sexual experiences (OSEs) involve a range of sex-related behaviors performed via the internet or social media, including accessing sexual information, exposure to graphic nudity, watching pornography, online dating, sexting, and naked chatting [4-6]. A study in Spain found a rising prevalence of sexting among high school students [7], and pornography use has been shown to be associated with sexually aggressive behaviors, such as sexual coercion, among American adults [8]. In the past few years, the COVID-19 pandemic has further increased OSEs as a result of decreased interpersonal physical intimacy due to social distancing measures [9].

In Chinese culture, sexuality is viewed more conservatively, and frequent parent-adolescent communication about sexuality has been shown to be associated with fewer sexual risk behaviors in young adults in mainland China [10]. However, a qualitative study found that family communication about sexuality was rare and implicit in mainland China [11]. A mixed-method study found that 87.3% of young adults in mainland China obtained sexual health information online [12]. In Hong Kong, a previous study found that one-tenth of young adults engaged in sexual risk behaviors [13]. Exposure to sexually explicit online materials was associated with subsequent psychological well-being and sexual permissive attitudes in Hong Kong adolescents [14]. Existing papers [4,7,8,12,14] only assessed specific subsets of OSEs, such as accessing sexual information, sexting, and watching pornography. The failure to acknowledge the diverse nature of OSEs implies that more research is necessary to investigate an inclusive list of OSEs.

Latent class analysis (LCA) is a statistical method that identifies latent classes based on respondents’ responses and elucidates diverse profiles of behavioral indicators [15]. Instead of using aggregated item averages, LCA can model the complexity and individual differences of OSEs. A literature search on Web of Science using the keywords of ((“sexual experience*” OR “sexual behavior*” OR “sexual activit*” [title]) AND latent class [title]) on April 6, 2023, found only 2 studies that explored OSEs using LCA, with small sample sizes (N=269-322) and analyses limited to female adolescents. These studies found 4 latent classes of OSEs, which were associated with sexual health and substance use [16,17]. However, they did not include young adults or males. A recent LCA study investigated sexual behavior indicators among youth in mainland China [18], but no existing papers have examined the latent heterogeneity of OSEs in young adults.

Given the importance of online sexual behaviors in health sciences [19], it is necessary to better understand the prevalence and profiles of OSEs among young adults. Multiple sex partners and unsafe sex have been shown to be associated with alcohol and tobacco use in Chinese youth [18] and American young adults [20], with sexual risk behaviors mediating sexual health outcomes [21,22]. However, no papers have examined the direct and indirect relationships between OSEs and behavioral health outcomes, namely, sexually transmitted infections (STIs), drug use, and suicidal ideation, via sexual risk behaviors and mental health. This study had the following 3 objectives: (1) to investigate the prevalence and heterogeneity of OSEs in young adults in Hong Kong; (2) to validate the derived OSE classes and analyze their associations with external variables, including demographic, family, psychological, and sex-related characteristics; and (3) to examine the direct and indirect relationships between OSE classes and behavioral health outcomes via sexual risk behaviors and mental health.

## Methods

### Study Design and Research Procedures

The data for this study originated from the 2021 Youth Sexuality Study (YSS) of the Hong Kong Family Planning Association, which was conducted every 5 years to examine sexual health and behaviors, and the psychosocial health of young adults. The sampling methods are described in the 2021 YSS survey report [23]. The survey was delayed due to the COVID-19 pandemic and was conducted via mobile phone from March to June 2022. The inclusion criteria were as follows: living in Hong Kong, age 18 to 27 years, and ability to understand spoken Cantonese or Putonghua. The survey was conducted by Social Policy Research Limited, a well-known third-party survey agency in Hong Kong. This agency has conducted a number of surveys on similar sensitive topics, such as organ donation, COVID-19 health-related risks, and fertility decisions, among various stakeholders in the general public. The agency has ample experience in undertaking studies of a similar nature with considerable expertise in survey planning and implementation, and data security. A total of 22,000 phone numbers were randomly generated, and a computer-assisted telephone interviewing system was used to contact potential respondents on weekdays from 6:30 PM to 10:30 PM.

The purpose and confidentiality of the survey were clearly explained to all potential participants, and voluntary participants provided oral informed consent. After excluding 1909 invalid telephone numbers and 17,253 numbers without target respondents, 2838 participants were eligible for the study. Some of the eligible participants did not take part in the phone interviews because they were too busy and could not make another survey appointment. In total, 1205 phone interviews were completed, with a response rate of 42.5%. Trained interviewers who were fluent in Cantonese and Putonghua conducted the interviews in around 20 minutes and provided emotional support information at the end. The survey did not collect personal identifying information and used anonymous questionnaires to encourage honest answers to sensitive questions, such as OSEs, sexual orientation, and sexual risk behaviors. The phone interviews were conducted in a private and secure setting. The interviewers used nonjudgmental and nonthreatening language to help participants feel comfortable and safe. Half of the interviews underwent quality checks by independent checkers, and 93.8% (563/600) of the sampled cases were successfully verified.

### Measures

The study questionnaire was designed by the 2021 YSS Task Force based on previous YSS questionnaires. It included questions on OSEs, demographic and family characteristics, mental health, sex-related factors, and behavioral health outcomes. It was field tested in the second half of 2021 and underwent minor revisions based on feedback from respondents and interviewers.

OSEs were assessed using 11 binary questions (yes/no) on lifetime experiences of online sex-related behaviors (Multimedia Appendix 1). The first 5 items were from the 2016 YSS, and the latter 6 were new items added to the 2021 YSS based on recent literature [7,24]. The 11 items referred to 3 types of OSEs [25]: nonarousal, solitary arousal, and partnered arousal. These items had good reliability (Cronbach α=.83) and satisfactory item-total correlations (*r*=0.40-0.62; *P*<.001) in the present sample.

Demographic characteristics assessed in the questionnaire included sex, age, education level, student status (versus working), and place of birth (Hong Kong or other places). Family characteristics included marital status (married versus single) and perceived family satisfaction via a single item “Are you happy with your family life?” on a 5-point Likert scale ranging from 1 (“very unhappy”) to 5 (“very happy”). Participants also reported the number of hours spent per week on social media.

The Patient Health Questionnaire-4 (PHQ-4) measured 4 anxiety and depression symptoms experienced by the participants over the past 2 weeks. The items were rated on a 4-point Likert scale ranging from 0 (“not at all”) to 3 (“nearly every day”), and the composite PHQ-4 score ranged from 0 to 12. The PHQ-4 has been validated as a brief assessment of psychological distress in Hong Kong young adults [26] and had good reliability (α=.86) in the present sample. Participants were asked about the perceived negative impact of COVID-19 on mental health on a 5-point Likert scale ranging from 1 (“much better”) to 5 (“much poorer”).

Questions on sex-related factors included sexual orientation (heterosexual=0 and nonheterosexual=1); sex desire (“How strong is your desire to have sex with others?” on a 5-point Likert scale ranging from 1 [“not at all”] to 5 [“very strong”]); and 12 knowledge statements on sex-related behaviors, HIV, and AIDS (response options of “true” or “false,” or “don’t know”). Each correct answer scored 1 point, and higher total scores (range: 0-12) indicated better sexual knowledge. The questionnaire assessed lifetime experiences of 5 sexual risk behaviors: sexually harassed others, sexual coercion (insisted on having sex while ignoring a partner’s wish), compensated dating with sex, unsafe sex without contraception, and multiple sex partners. The items on sexual knowledge and sexual risk behaviors showed good reliability (α=.74-.83) in the present sample.

The study assessed 3 behavioral health outcomes, namely, STIs, drug use, and suicidal ideation, of the participants in the past year. Drug use referred to the use of various psychotropic substances such as ketamine, ecstasy, cocaine, and heroin.

### Statistical Analysis

The variables were summarized using descriptive statistics, and the reliabilities of OSEs, PHQ-4 scores, sexual knowledge, and sexual risk behaviors were assessed via Cronbach α coefficients. Sampling weights were calculated based on 2021 population census data stratified by sex, place of birth, and age group to correct for the imbalance in the sample. Weighted analyses were used for all subsequent analyses unless otherwise stated. Missing data were handled using full information maximum likelihood under the missing-at-random assumption [27]. LCA was used to identify latent classes based on the 11 OSE items among young adults. LCA models with 1 to 5 classes were estimated using the robust maximum likelihood estimator in Mplus 8.6 [28]. Model fit was evaluated using the Bayesian information criterion (BIC), with lower values indicating better fit. The Lo-Mendell-Rubin (LMR) likelihood ratio test [29] compared the fit of the k-class LCA model to the alternative k-1 class model, with a small *P*-value of <.01 favoring the former. Model classification quality was assessed using entropy and average latent class probabilities, with high values (>0.90) indicating adequate classification.

The heterogeneity of the OSEs in the sample was described via prevalence and conditional item probabilities of latent classes, with probabilities above 0.40 considered substantial. LCA models were estimated separately for males and females to test the stability of the latent class structure by sex. Measurement invariance was evaluated by comparing the BIC of the LCA models with and without equality constraints on the OSE item thresholds. Latent classes were checked against demographic and family characteristics, mental health, sex-related factors, and behavioral health outcomes. Class differences were estimated using the Bolck, Croon, and Hagenaars (BCH) procedure [30], with post-hoc comparisons using Sidak correction. Sidak correction controlled the family-wise error rate with slightly higher power than the Bonferroni correction method. Multinomial logistic regression with a 3-step approach used demographic and family characteristics and sex-related factors as predictors of latent class membership, with the strengths of associations estimated by adjusted odds ratios (ORs).

We used a structural equation model (SEM) to investigate the associations among OSE latent classes, sexual risk behaviors, PHQ-4 scores, and behavioral health outcomes. The most likely OSE class membership was the primary independent variable; sexual risk behaviors and PHQ-4 scores were latent mediators; and STIs, drug use, and suicidal ideation were binary outcomes. Model fit was evaluated by the following criteria on fit indices: root mean square error of approximation (RMSEA) ≤0.06, comparative fit index (CFI) ≥0.95, and standardized root mean square residual (SRMR) ≤0.06. The SEM estimated standardized regression coefficients for direct (β) and indirect effects (αβ) of the OSE latent class on behavioral health outcomes via the mediators. The SEM included covariates, namely, sex, age, place of birth, education level, student status, marital status, family satisfaction, sexual orientation, sexual knowledge, COVID-19 mental impact, and time spent on social media. The direct and indirect effects were estimated using bootstrapping with 10,000 bootstrap draws. Statistical significance was determined by 95% CIs not including zero for the direct and indirect effects, and 95% CIs not including one for the ORs.

### Ethical Considerations

The study received ethical approval from the Human Research Ethics Committee of the University of Hong Kong (reference number: EA200333). All participants provided oral informed consent after the survey’s purpose was explained, with anonymity, confidentiality, and voluntary participation emphasized. The privacy of personal information was protected throughout the study via anonymous data collection, and confidentiality was preserved to ask participants to provide honest answers. Eligible participation in this survey was voluntary and was not compensated. The authors assert that all procedures contributing to this work comply with the ethical standards of the relevant national and institutional committees on human experimentation and with the Helsinki Declaration of 1975, as revised in 2008.

## Results

### Sample Characteristics

Table 1 shows that among 1205 participants (613 males and 592 females), the average age was 23.0 (SD 2.86) years. The majority of them were born in Hong Kong, working, single, and heterosexual. They had good sexual knowledge (mean 10.1, SD 2.19) and spent a mean of 2.94 (SD 2.04) hours per day on social media. The prevalence of sexual risk behaviors and behavioral health outcomes ranged from 3.2% (38/1205) to 12.3% (148/1205) and from 1.7% (20/1205) to 6.5% (78/1205), respectively. As shown in Multimedia Appendix 1, more than half (616/1205, 51.1% to 801/1205, 66.5%) of the participants were exposed to pornographic content, accessed sexual content, and discussed sex with others online or on social media. Naked chatting online, sending pornographic messages online, and having sex with people acquainted with online or on social media had the lowest prevalence (77/1205, 6.4% to 134/1205, 11.1%). Male participants showed higher prevalence of all 11 OSEs (χ^2^_1_=20.6-55.7; *P*<.001) than female participants.

**Table 1:** Demographic and family characteristics, mental health data, sex-related factors, and behavioral health outcomes.

Variable			Value (N=1205)
Female sex, n (%)			592 (49.1)
**Education level, n (%)**			
	Secondary school	389 (32.3)	
	Associate degree	266 (22.1)	
	Bachelor’s degree or above	551 (45.7)	
**Current status, n (%)**			
	Student	478 (39.7)	
	Working	727 (60.3)	
Born in Hong Kong, n (%)			894 (74.2)
**Marital status, n (%)**			
	Married	149 (12.4)	
	Single	1056 (87.6)	
**Sexual status, n (%)**			
	Heterosexual	1117 (92.7)	
	Nonheterosexual	88 (7.3)	
**Sexual risk behaviors, n (%)**			
	Sexually harassed others	61 (5.1)	
	Sexual coercion	66 (5.5)	
	Compensated dating with sex	38 (3.2)	
	Unsafe sex	148 (12.3)	
	Multiple sex partners	82 (6.8)	
**Behavioral health outcomes, n (%)**			
	Sexually transmitted infections	20 (1.7)	
	Drug use	40 (3.3)	
	Suicidal ideation	78 (6.5)	
Age (years), mean (SD; range)			23.0 (2.86; 18-27)
Family satisfaction^a^, mean (SD; range)			3.60 (0.79; 1-5)
Daily hours spent on social media, mean (SD; range)			2.94 (2.04; 0-8)
COVID-19 mental impact^a^, mean (SD; range)			3.24 (0.86; 1-5)
Psychological distress^a^ (PHQ-4^b^), mean (SD; range)			2.17 (2.34; 0-12)
Sex desire^a^, mean (SD; range)			3.14 (0.93; 1-5)
Sexual knowledge^a^, mean (SD; range)			10.1 (2.19; 0-12)

^a^Higher scores indicate better family satisfaction, more negative COVID-19 mental impact, and higher levels of psychological distress, sex desire, and sexual knowledge.

^b^PHQ-4: Patient Health Questionnaire-4.

### Latent Class Models

Multimedia Appendix 2 shows a decreasing trend in the BIC from 1-class to 5-class LCA models in the whole sample and in males and females separately, with substantial decreases in the BIC from 1-class to 3-class models. The LMR likelihood ratio tests did not show significant improvement (*P*=.10-.38) in model fit for the 4-class model over the 3-class model. These findings supported the 3-class LCA model with good entropy and average latent class probabilities. Multimedia Appendices 3 and 4 show the class prevalence and conditional item probabilities of the 3-class LCA model in males and females, respectively. The multiple-group LCA model with sex-invariant item thresholds showed a substantially lower BIC (12,792 versus 12,925) than the noninvariant LCA model, supporting measurement invariance of the latent class structure across sex.

As shown in Table 2, the abstinent class comprised 33.1% (398/1205) of participants and had the lowest conditional item probabilities (1/398, 0.3% to 46/398, 11.5%) of all OSEs. The normative class, comprising 56.0% (675/1205) of participants, accessed sexual content and pornographic content online or on social media, discussed sex with others on social media, and sought pornographic content online actively. This class did not show substantial prevalence (<40%) in the remaining 7 items. The active class, comprising 10.9% (132/1205) of participants, had the highest conditional item probabilities (65/132, 49.5% to 132/132, 100%) of all OSEs. The prevalences of the normative and active classes were higher among males (378/613, 61.6% and 104/613, 17.0%, respectively) than among females (301/592, 50.9% and 28/592, 4.7%, respectively).

**Table 2:** Latent class prevalence of the 3-class model with measurement invariance across sex and the conditional item probabilities of online sexual experiences.

Variable			Class 1: abstinent (N=398)		Class 2: normative (N=675)		Class 3: active (N=132)
**Latent class prevalence, n (%)**							
	Whole sample (N=1205)	398 (33.1)		675 (56.0)		132 (10.9)	
	Males (N=613)	131 (21.4)		378 (61.6)		104 (17.0)	
	Females (N=592)	263 (44.4)		301 (50.9)		28 (4.7)	
**Item^a^, n (%)**							
	2. Exposed to pornographic content online or on social media	30 (7.6)		637 (94.4)^b^		131 (99.2)^b^	
	6. Accessed sexual content online or on social media	15 (3.7)		558 (82.7)^b^		132 (100.0)^b^	
	1. Discussed sex with others on social media	46 (11.5)		441 (65.3)^b^		126 (95.1)^b^	
	9. Actively sought pornographic content online or on social media	12 (2.9)		328 (48.6)^b^		124 (94.0)^b^	
	3. Dated people acquainted with online or on social media	28 (7.1)		229 (34.0)		119 (89.9)^b^	
	11. Exposed to pornographic content in internet games	10 (2.6)		227 (33.6)		103 (77.7)^b^	
	4. Received pornographic (text or video) messages online	7 (1.7)		186 (27.6)		81 (61.4)^b^	
	10. Posted/shared indecent photos online or on social media	2 (0.4)		103 (15.2)		100 (76.1)^b^	
	7. Had sex with people acquainted with online or on social media	2 (0.5)		45 (6.7)		86 (65.3)^b^	
	5. Sent pornographic (text or video) messages online	1 (0.3)		53 (7.9)		66 (49.9)^b^	
	8. Had naked chat online	2 (0.4)		9 (1.4)		65 (49.5)^b^	

^a^Items are presented in descending order for the crude unweighted probabilities. The item numbers indicate the order in the questionnaire.

^b^Substantial conditional item probabilities that are greater than 0.40 for the latent classes.

### Comparison of Profiles Across the Latent Classes

As shown in Table 3, females had a significantly lower prevalence of the normative and active classes than the abstinent class. No significant differences (χ^2^_2_=2.17-4.68; *P*=.10-.34) were found in age, place of birth, education level, student status, and marital status. Compared with the abstinent class, the normative class had significantly higher sex desire and PHQ-4 scores, while the active class had significantly lower family satisfaction and spent more time on social media, and both the normative and active classes had a significantly more negative COVID-19 mental impact and higher rates of suicidal ideation. The normative class had significantly higher sexual knowledge and lower unsafe sex rates than the other 2 classes. The active class had significantly higher sex desire and PHQ-4 scores and higher rates of nonheterosexual orientation, other sexual risk behaviors, STIs, and drug use than the other 2 classes. For instance, young adults in the active class had significantly higher rates of sexually harassed others, sexual coercion, compensated dating with sex, and multiple sex partners than those in the normative class or abstinent class.

**Table 3:** Demographic and family characteristics, mental health, sex-related factors, and behavioral health outcomes of the 3 latent classes of online sexual experiences (N=1205).

Variables	Abstinent class (N=398, 33.1%)	Normative class (N=675, 56.0%)	Active class (N=132, 10.9%)	Overall 3-class difference, χ^2^ (*df*)	*P* value
Female sex, n (%)	264 (66.4)^a^	300 (44.4)^b^	28 (21.3)^c^	88.9 (2)	<.001
Age (years), mean (SE)	22.7 (0.17)	23.1 (0.12)	23.2 (0.30)	4.60 (2)	.10
Born in Hong Kong, n (%)	287 (72.0)	497 (73.7)	1111 (83.9)	4.68 (2)	.10
Education level, mean (SE)	2.08 (0.05)	2.17 (0.04)	2.13 (0.09)	2.17 (2)	.34
Current student status, n (%)	176 (44.1)	254 (37.7)	48 (36.7)	3.64 (2)	.16
Married status, n (%)	39 (9.9)	97 (14.4)	13 (10.0)	3.58 (2)	.17
Family satisfaction (1-5)^d^, mean (SE)	3.68 (0.04)^b^	3.60 (0.03)^b,c^	3.38 (0.09)^c^	8.76 (2)	.01
Nonheterosexual, n (%)	16 (3.9)^c^	45 (6.7)^c^	26 (19.9)^b^	16.3 (2)	<.001
Sexual knowledge (0-12)^d^, mean (SE)	9.39 (0.15)^c^	10.5 (0.08)^b^	9.83 (0.26)^c^	46.3 (2)	<.001
Sex desire (1-5)^d^, mean (SE)	2.62 (0.06)^c^	3.30 (0.04)^b^	3.76 (0.11)^a^	143.6 (2)	<.001
COVID-19 mental impact (1-5)^d^, mean (SE)	3.05 (0.05)^c^	3.35 (0.04)^b^	3.30 (0.09)^b^	24.7 (2)	<.001
Daily hours on social media, mean (SE)	2.69 (0.11)^c^	2.98 (0.09)^b,c^	3.51 (0.20)^b^	13.3 (2)	.001
Sexually harassed others, n (%)	6 (1.6)^c^	18 (2.7)^c^	38 (28.5)^b^	37.3 (2)	<.001
Sexual coercion, n (%)	11 (2.8)^c^	24 (3.5)^c^	32 (24.0)^b^	23.0 (2)	<.001
Compensated dating with sex, n (%)	0 (0.0)^c^	18 (2.7)^b^	21 (15.6)^a^	36.1 (2)	<.001
Unsafe sex, n (%)	70 (17.7)^b^	59 (8.7)^c^	27 (20.2)^b^	7.89 (2)	.02
Multiple sex partners, n (%)	8 (2.1)^c^	37 (5.5)^c^	36 (27.4)^b^	36.9 (2)	<.001
Psychological distress (PHQ-4^e^) (0-12)^d^, mean (SE)	1.44 (0.11)^c^	2.36 (0.10)^b^	3.39 (0.34)^a^	59.3 (2)	<.001
Sexually transmitted infections, n (%)	3 (0.8)^c^	7 (1.1)^c^	10 (7.8)^b^	7.40 (2)	.03
Drug use, n (%)	3 (0.7)^c^	21 (3.1)^b^	17 (12.6)^a^	19.3 (2)	<.001
Suicidal ideation, n (%)	8 (2.1)^c^	50 (7.4)^b^	20 (15.0)^b^	25.5 (2)	<.001

^a,b,c^Significant post-hoc differences among the 3 latent classes (c < b < a).

^d^Higher scores indicate better family satisfaction, more negative COVID-19 mental impact, and higher levels of psychological distress, sexual knowledge, and sex desire.

^e^PHQ-4: Patient Health Questionnaire-4.

Age, place of birth, education level, current student, and marital status were not significantly associated (*P=*.13–.98) with the latent class membership (Table 4). Females had low odds of the active class (OR 0.19, 95% CI 0.09-0.38; *P*<.001) compared with the other 2 classes. Higher COVID-19 mental impact (OR per score 1.54, 95% CI 1.23-1.89; *P*<.001) was associated with high odds of the normative class compared with the abstinent class. Family satisfaction was associated with low odds of the active class (OR per score 0.67-0.68; *P*=.03-.049) compared with the other 2 classes, while nonheterosexual orientation (OR 3.36-6.82; *P*<.001) and daily hours spent on social media (OR 1.13-1.19; *P*=.01-.04) were associated with high odds of the active class compared with the other 2 classes. Sexual knowledge was associated with high odds of the normative class compared with the other 2 classes (OR 1.16-1.29; *P=*.001-.006).

**Table 4:** Multinomial logistic regression for associations of the latent class memberships of online sexual experiences with demographic, family, psychological, and sex-related factors (N=1123).

Variable	Active class (reference: abstinent)		Normative class (reference: abstinent)		Active class (reference: normative)	
	OR^a^ (95% CI)	*P* value	OR (95% CI)	*P* value	OR (95% CI)	*P* value
Female (versus male)	0.19 (0.09-0.38)	<.001	0.49 (0.34-0.71)	<.001	0.38 (0.20-0.75)	.005
Age, per year	1.03 (0.89-1.20)	.66	0.99 (0.90-1.09)	.81	1.05 (0.91-1.20)	.51
Born in Hong Kong (versus other places)	1.84 (0.84-4.06)	.13	1.13 (0.70-1.83)	.61	1.62 (0.78-3.38)	.20
Education level (1-3)^b^	1.01 (0.70-1.45)	.96	1.02 (0.81-1.27)	.90	1.00 (0.71-1.39)	.98
Current student (versus working)	0.97 (0.41-2.31)	.95	0.82 (0.47-1.43)	.49	1.18 (0.53-2.64)	.69
Married (versus single)	0.62 (0.26-1.48)	.28	1.01 (0.58-1.74)	.98	0.61 (0.27-1.37)	.23
Family satisfaction (1-5)^b^	0.68 (0.47-0.99)	.049	1.02 (0.81-1.29)	.87	0.67 (0.47-0.95)	.03
Nonheterosexual (versus heterosexual)	6.82 (2.32-20.1)	<.001	2.03 (0.85-4.83)	.11	3.36 (1.50-7.53)	.003
Sexual knowledge (0-12)^b^	1.10 (0.98-1.23)	.09	1.29 (1.18-1.40)	<.001	0.86 (0.77-0.96)	.006
Sex desire (1-5)^b^	3.69 (2.51-5.42)	<.001	2.22 (1.80-2.73)	<.001	1.66 (1.16-2.39)	.006
COVID-19 mental impact (1-5)^b^	1.37 (0.99-1.89)	.06	1.54 (1.23-1.89)	<.001	0.89 (0.67-1.19)	.45
Daily hours on social media, per hour	1.19 (1.04-1.35)	.01	1.05 (0.96-1.15)	.30	1.13 (1.01-1.27)	.04

^a^OR: odds ratio.

^b^For continuous independent variables, ORs are per score. Higher scores indicate better family satisfaction, more negative COVID-19 mental impact, and higher levels of psychological distress, sexual knowledge, and sex desire.

### Associations Between the Active Class and Behavioral Health Outcomes

Considering the higher rates of behavioral health outcomes in the active class than in the nonactive classes, the SEM was used to examine the effects of the active class on the behavioral health outcomes. The SEM fitted the data approximately well (RMSEA=0.026, CFI=0.98, SRMR=0.056). Significant factor loadings were found for sexual risk behaviors and PHQ-4 scores. Male sex, being married, nonheterosexual orientation, and time spent on social media were associated (β=0.13-0.27; *P=*.001-.02) with more sexual risk behaviors. Female sex, younger age, education level, being married, lower family satisfaction, nonheterosexual orientation, and higher COVID-19 mental impact were associated (β=0.09-0.28; *P=*.001-.04) with higher PHQ-4 scores. COVID-19 mental impact was positively associated (β=0.18-0.27; *P=*.001-.02) with STIs, drug use, and suicidal ideation.

**Figure 1:**
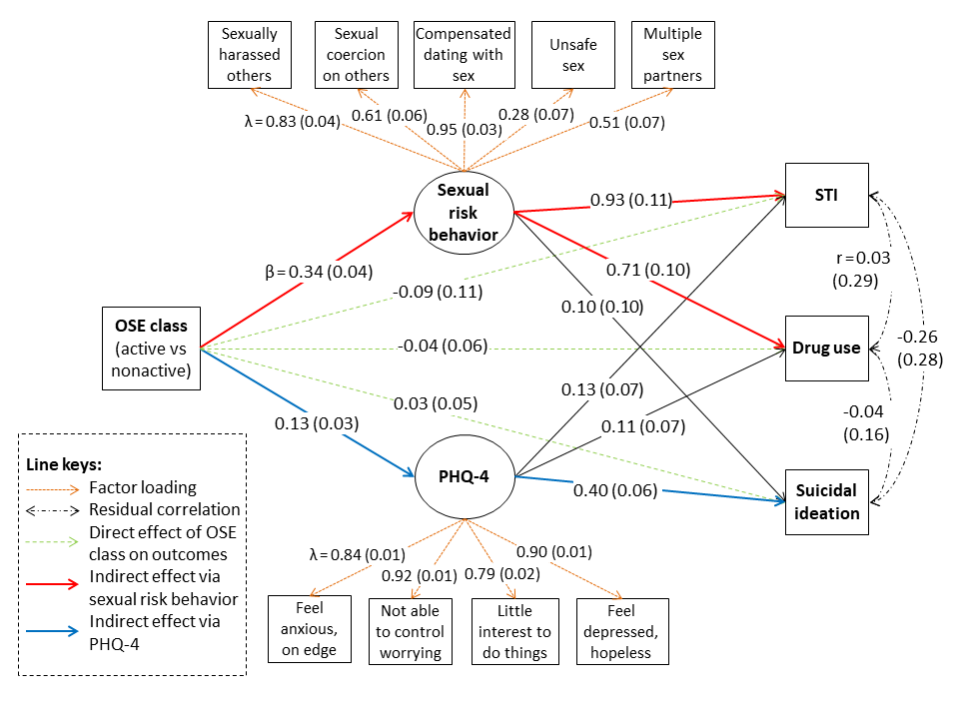
Standardized coefficients in the structural equation model on the associations among the active class (N=132) compared with the nonactive (abstinent and normative) classes (N=1075) of online sexual experiences (OSEs), sexual risk behaviors, Patient Health Questionnaire-4 (PHQ-4) scores, sexually transmitted infections (STIs), drug use, and suicidal ideation. For simplicity of presentation, model covariates and residual correlations between sexual risk behaviors and PHQ-4 scores are not shown. Standard errors of the coefficients are shown in parenthesis. Factor loadings (λ) of the latent factors of sexual risk behaviors and PHQ-4 scores are shown with orange dotted arrows. The direct effects (β) of OSE class on STIs, drug use, and suicidal ideation are shown with pale green dashed arrows. The indirect effects of OSE class on STIs and drug use via sexual risk behaviors are highlighted with red solid arrows. The indirect effects of OSE class on suicidal ideation via PHQ-4 scores are highlighted with blue solid arrows. The residual correlations (r) among STIs, drug use, and suicidal ideation are shown with black dash-dotted arrows. Significant indirect effects (red and blue solid arrows) are highlighted in bold.

Figure 1 shows the SEM results. The model covariates and residual correlations between sexual risk behaviors and PHQ-4 scores have not been shown for simplicity. Controlling for the model covariates, the active class was positively associated with sexual risk behaviors and PHQ-4 scores (β=0.13-0.34; *P*<.001). Sexual risk behaviors were positively associated with STIs and drug use (β=0.71-0.93; *P*<.001), and PHQ-4 scores were significantly positively associated with suicidal ideation (β=0.40; *P*<.001). The residual correlations among the 3 behavioral health outcomes were not statistically significant (*r*=−0.26 to 0.03; *P=*.35-.91). There was a positive residual correlation between sexual risk behaviors and PHQ-4 scores (*r*=0.28; *P*<.001).

The active class did not have significant direct effects (β=−0.09 to 0.03; *P*=.41-.62) on behavioral health outcomes. However, the active class had positive indirect effects on STIs (αβ=0.31; *P*<.001) via sexual risk behaviors, drug use (αβ=0.24; *P*<.001) via sexual risk behaviors, and suicidal ideation (αβ=0.05; *P*<.001) via PHQ-4 scores. These results implied that sexual risk behaviors substantially mediated the effects of the active class on STIs and drug use, and PHQ-4 scores substantially mediated the effects of the active class on suicidal ideation. The model explained 48.8%, 52.7%, and 81.7% of the variances of suicidal ideation, drug use, and STIs, respectively. Compared with the nonactive classes, the active class was indirectly associated with higher prevalences of STIs (Δ [absolute difference in percentage points]=4.8%, 95% CI 0.5%-8.1%), drug use (Δ=7.6%, 95% CI 2.9%-11.8%), and suicidal ideation (Δ=2.5%, 95% CI 0.8%-4.3%).

## Discussion

### Principal Findings

This is the first study to report results on the latent heterogeneity of OSEs in young adults. Young adults could be classified into the following 3 latent classes: abstinent class with minimal OSEs, normative class with occasional OSEs, and active class with substantive OSEs. The measurement invariance test results supported stability of the 3 classes across sex. The 3 derived OSE classes were validated with distinct profiles in demographic, family, psychological, and sex-related characteristics amid the COVID-19 pandemic in 2022. Moreover, the SEM found significant indirect associations between OSE classes and STIs, drug use, and suicidal ideation via sexual risk behaviors and mental health. In particular, sexual risk behaviors were found to mediate the relationships between OSE classes and STIs and drug use, and mental health was found to mediate the relationship between OSE classes and suicidal ideation.

### Comparison With Prior Work

The normative class, comprising the majority of the sample, had mainly nonarousal and solitary arousal OSEs, indicating passive exposure of sex-related information and active pursuit of sex knowledge. This class showed higher sexual knowledge but not increased sexual risk behaviors compared with the other classes and could represent young adults who are curious about sexuality and explore pornographic content online. A cluster randomized controlled trial on internet-based sexuality education programs significantly improved the sexual knowledge of adolescents in mainland China [31]. Our 2021 YSS survey found that internet use was positively associated with sexual knowledge in Hong Kong adolescents amid the COVID-19 pandemic [32]. A national survey in Germany found greater perceived positive outcomes of OSEs (eg, better sexual well-being) than negative outcomes (eg, online dating scams and cyberbullying) in adults [33]. Our findings align with these results and suggest a beneficial relationship between implicit types of OSEs and better sexual knowledge in young adults.

The prevalence of our normative and active classes (66.9%) was similar to the prevalence of online sexual activities (68%) in a previous German study [33]. In our study, males showed a lower prevalence of the abstinent class and a much higher prevalence of the active class than females. This sex difference is consistent with the findings of a meta-analysis [34] on the higher prevalence of online sexual behaviors among young male adults and the literature on higher sensation-seeking and lower impulse control in males in early adulthood [35]. In our sample, there was a significantly higher sex desire (Cohen *d*=0.71; *P*<.001) in males than in females. A longitudinal study on British young adults found significant decreases in sex desire in females but not males during COVID-19 social lockdown restrictions compared with prelockdown levels [36]. Future studies should examine sex differences in OSEs across cultural contexts.

We found that the active class had higher PHQ-4 scores and more sexual risk behaviors than the nonactive classes. These findings agree with previous studies on the positive associations between sexting and depressive symptoms in American youth [4] and between pornography use and sexual risk behaviors in Chinese youth [18]. All of the 3 residual correlations among STIs, drug use, and suicidal ideation were not statistically significant in the SEM. This suggests that the behavioral health outcomes were relatively well explained by the OSE class and latent mediators such that there were no leftover associations among them. The active class was at risk of behavioral health outcomes via sexual risk behaviors and PHQ-4 scores, and our results suggest a mediating role for sexual risk behaviors in the relationship of the active class with STIs and drug use. We recently found a mediating role for PHQ-4 scores between meaning in life and suicidal ideation in Hong Kong young adults [26]. PHQ-4 could be a simple and useful tool in future studies on the relationship between OSEs and risk factors.

This study has several strengths. First, we developed 11 items to measure 3 types of OSEs in young adults [25]. The moderate item-total correlations indicated good reliability and adequate discriminant validity of the items. Second, LCA classified the participants from empirical data into distinct latent classes of OSEs while allowing for potential misclassification errors. The 3 latent classes were validated using the BCH procedure and a 3-step approach [30]. Third, the SEM offered more reliable results by accounting for measurement errors of the latent factors and estimated the direct and indirect effects of OSE classes on behavioral health outcomes.

Early prevention and intervention are necessary to reduce the health burdens on young adults and society. The active class, although the smallest group, had more nonheterosexual individuals, had a worse family relationship, and spent more time on social media than the nonactive classes. Recent studies [37,38] found higher rates of sexual and suicidal risks in nonheterosexual individuals among Canadian and American young adults. Future research can explore risk factors like HIV knowledge and sexual identity stigma in this group [39-41]. Our previous study found that lower family satisfaction was associated with more sexual risk behaviors in Hong Kong adolescents [42]. The COVID-19 pandemic has increased young adults’ social media use, and those with a poor family relationship may rely on the internet for sexual content and be exposed to pornographic content online. Greater internet exposure has been shown to be associated with sexual risk behaviors among young male adults in mainland China [43].

### Limitations

Our study had several limitations. First, the cross-sectional design made it difficult to determine the directionality between OSE classes and other variables. Since both OSEs and sexual risk behaviors were measured over the lifetime, their relationships could be bidirectional with reciprocal effects from sexual risk behaviors to greater OSEs. Future longitudinal studies are needed to clarify the temporal relationships among OSEs, sexual risk behaviors, mental health, and behavioral health outcomes. Second, we did not assess the underlying causes for OSEs, and young adults could engage in OSEs for different purposes such as boredom regulation, sexual gratification, and sensation seeking. Further qualitative studies should elucidate and compare the associations and motives for OSEs in the normative and active classes. Third, we did not differentiate between active solicitation and passive exposure in some OSE items, which may have included unwanted or forced behaviors. Further studies should examine the relationships between unwanted OSEs and online sexual harassment. Fourth, our questionnaire did not consider potential confounding factors such as impulsivity, compulsivity, internet and gaming addictions, and peer influences. Future research could examine the comorbidity between problematic OSEs and other risk or protective factors via network analysis. Fifth, the 11 OSE items we developed were not previously validated. Future studies should develop a new scale on OSEs with reference to our items and evaluate the psychometric properties. Sixth, the response rate of the phone survey was only 42.5%, and over half of the eligible participants did not take part in the phone interviews. The relatively low response rate implied potential selection biases. Our study sample of 1205 interviewed people might differ from the 1633 noninterviewed people in terms of OSEs and sexual risk behaviors. It was plausible that young adults with active OSEs could opt out of the survey, which led to underreporting of OSEs and a reduction in the effect size. It is necessary to be cautious when generalizing the present results to the general population of young adults.

### Conclusions and Implications

This study provides the first results on the 3 latent classes of OSEs (abstinent, normative, and active) with distinct profiles in demographic and family characteristics, mental health, sex-related factors, and behavioral health outcomes. The active class showed indirect associations with higher rates of STIs and drug use via sexual risk behaviors and higher rates of suicidal ideation via PHQ-4 scores than the nonactive classes. Our results have clinical implications for personalized interventions to help young adults with substantive OSEs.

In Hong Kong, school-based sex education primarily focuses on the biological aspects of sexual and reproductive health among young adults, and sex education could promote sexual knowledge and be protective against STIs [44]. Comprehensive sex education interventions in northwest China have been successful in increasing sexual knowledge and sexual self-efficacy among adolescents [45]. Recent reviews [46-48] suggest that sex education programs should emphasize the social aspects of sexual health via positive youth development approaches and include up-to-date topics, such as internet pornography, safe use of social media, and caveats of OSEs, to promote media literacy. Social media platforms could be an effective means of delivering sex education programs, and a pilot randomized controlled trial [49] in the United States has shown feasibility in reducing sexual risk behaviors and drug use via mobile health apps among 50 young individuals. Future research is needed to test the cost-effectiveness of mobile health apps in promoting sex knowledge, enhancing self-efficacy for refusing risky OSEs, reducing sexual risk behaviors, and preventing STIs and drug use among young adults.

## Appendix

Multimedia Appendix 1 Prevalence of online sexual experiences in male and female participants.

Multimedia Appendix 2 Fit indices and classification quality of latent class models on online sexual experiences in the whole sample and in male and female participants separately.

Multimedia Appendix 3 Latent class prevalence of the 3-class model without measurement invariance across sex and the conditional item probabilities of online sexual experiences in male participants.

Multimedia Appendix 4 Latent class prevalence of the 3-class model without measurement invariance across sex and the conditional item probabilities of online sexual experiences in female participants.

## Abbreviations

BCH: Bolck, Croon, and Hagenaars
BIC: Bayesian information criterion
CFI: comparative fit index
LCA: latent class analysis
LMR: Lo-Mendell-Rubin
OR: odds ratio
OSE: online sexual experience
PHQ-4: Patient Health Questionnaire-4
RMSEA: root mean square error of approximation
SEM: structural equation model
SRMR: standardized root mean square residual
STI: sexually transmitted infection
YSS: Youth Sexuality Study

## Data Availability

The raw data analyzed in this study are available from the first author (TCTF).
