# Supplementary material for: Latent Heterogeneity of Online Sexual Experiences and Associations With Sexual Risk Behaviors and Behavioral Health Outcomes in Chinese Young Adults: Cross-Sectional Study
Source: JMIR Public Health Surveill. 2024 Jan 26;10:e50020. doi: 10.2196/50020 (PMC10858424; doi:10.2196/50020)
Supplement: Multimedia Appendix 1 [file publichealth_v10i1e50020_app1.docx]

| **Multimedia Appendix 1.** Prevalence of online sexual experiences in male and female participants. | | | | |
| --- | --- | --- | --- | --- |
|  | Unweighted estimates | | |  |
| Online sexual experiences (yes %): | Whole sample | Male | Female | χ^2^ |
| 1. Discussed sex with others on social media | 51.1% | 60.7% | 41.0% | 46.5*** |
| 2. Exposed to pornographic content online or on social media | 66.5% | 76.0% | 56.7% | 50.5*** |
| 3. Dated people acquainted with online or on social media | 31.3% | 37.7% | 24.8% | 23.1*** |
| 4. Received pornographic (text or video) messages online | 23.1% | 32.0% | 13.9% | 55.7*** |
| 5. Sent pornographic (text or video) messages online | 10.1% | 15.0% | 5.1% | 32.5*** |
| 6. Accessed sexuality content online or on social media | 58.7% | 66.9% | 50.2% | 34.7*** |
| 7. Had sex with people acquainted with online or on social media | 11.1% | 15.1% | 6.9% | 20.6*** |
| 8. Had naked chat online | 6.4% | 9.8% | 2.7% | 25.5*** |
| 9. Actively sought pornographic content online or on social media | 38.8% | 49.1% | 28.2% | 55.3*** |
| 10. Posted/shared indecent photos online or on social media | 17.0% | 22.3% | 11.5% | 25.2*** |
| 11. Exposed to pornographic content in internet games | 28.4% | 36.2% | 20.3% | 37.7*** |
| *** *P*<.001; N = 1205/582/623 for whole sample/males/females. The items are presented in the order of the questionnaire. | | | | |
