# Supplementary material for: Latent Heterogeneity of Online Sexual Experiences and Associations With Sexual Risk Behaviors and Behavioral Health Outcomes in Chinese Young Adults: Cross-Sectional Study
Source: JMIR Public Health Surveill. 2024 Jan 26;10:e50020. doi: 10.2196/50020 (PMC10858424; doi:10.2196/50020)
Supplement: Multimedia Appendix 2 [file publichealth_v10i1e50020_app2.docx]

| **Multimedia Appendix 2.** Fit indices and classification quality of latent class models on online sexual experiences in the whole sample and in male and female participants separately. | | | | | |
| --- | --- | --- | --- | --- | --- |
| Model | # | BIC | LMR *P*-value | Entropy | Average latent class probabilities |
| Whole sample (N = 1,205) |  |  |  |  |  |
| 1-class | 11 | 14066 | / | / | / |
| 2-class | 23 | 11799 | <.001 | .87 | .95 – .98 |
| 3-class | 35 | 11261 | <.001 | .89 | .90 – .97 |
| 4-class | 47 | 11156 | .38 | .80 | .84 – .96 |
| 5-class | 59 | 11074 | .15 | .85 | .84 – .97 |
| Males (N = 613) |  |  |  |  |  |
| 1-class | 11 | 7277 |  |  |  |
| 2-class | 23 | 6417 | <.001 | .79 | .90 – .96 |
| 3-class | 35 | 6094 | <.001 | .89 | .95 – .96 |
| 4-class | 47 | 6032 | .19 | .88 | .90 – .94 |
| 5-class | 59 | 5992 | .09 | .84 | .87 – .96 |
| Females (N = 592) |  |  |  |  |  |
| 1-class | 11 | 6366 | / | / | / |
| 2-class | 23 | 5203 | <.001 | .92 | .97 – .98 |
| 3-class | 35 | 5106 | <.001 | .88 | .92 – .98 |
| 4-class | 47 | 5094 | .10 | .88 | .85 – .97 |
| 5-class | 59 | 5116 | .63 | .88 | .83 – .99 |
| # = number of free parameters; BIC = Bayesian information criterion; LMR = Lo-Mendell-Rubin likelihood ratio test. Lower values of BIC and LMR (*P*<.001) indicate better model fit and higher values of entropy and average latent class probabilities indicate better model classification. | | | | | |
