# Supplementary material for: Latent Heterogeneity of Online Sexual Experiences and Associations With Sexual Risk Behaviors and Behavioral Health Outcomes in Chinese Young Adults: Cross-Sectional Study
Source: JMIR Public Health Surveill. 2024 Jan 26;10:e50020. doi: 10.2196/50020 (PMC10858424; doi:10.2196/50020)
Supplement: Multimedia Appendix 3 [file publichealth_v10i1e50020_app3.docx]

| **Multimedia Appendix 3.** Latent class prevalence of the 3-class model without measurement invariance across sex and the conditional item probabilities of online sexual experiences in male participants. | | | |
| --- | --- | --- | --- |
| Item probabilities of online sexual experiences (yes %): | Class 1: Abstinent  (N = 146) | Class 2: Normative  (N = 361) | Class 3: Active  (N = 75) |
| Latent class prevalence: | 25.0% | 62.0% | 13.0% |
| 2. Exposed to pornographic content online or on social media | 18.6% | **94.4%** | **98.8%** |
| 6. Accessed sexuality content online or on social media | 10.9% | **82.6%** | **100.0%** |
| 1. Discussed sex with others on social media | 19.0% | **70.0%** | **96.6%** |
| 9. Actively sought pornographic content online or on social media | 6.2% | **56.6%** | **95.5%** |
| 3. Dated people acquainted with online or on social media | 6.1% | 37.3% | **100.0%** |
| 11. Exposed to pornographic content in internet games | 1.2% | **40.7%** | **81.8%** |
| 4. Received pornographic (text or video) messages online | 3.4% | 38.3% | **56.6%** |
| 10. Posted/shared indecent photos online or on social media | 0.5% | 18.9% | **81.0%** |
| 7. Had sex with people acquainted with online or on social media | 0.8% | 6.1% | **85.8%** |
| 5. Sent pornographic (text or video) messages online | 0.5% | 13.4% | **49.9%** |
| 8. Had naked chat online | 0.6% | 2.8% | **61.1%** |
| N = 582; Substantial conditional item probabilities that are greater than .40 are bolded for the latent classes. Items were presented in descending order for the crude unweighted probabilities. | | | |
