# Supplementary material for: Latent Heterogeneity of Online Sexual Experiences and Associations With Sexual Risk Behaviors and Behavioral Health Outcomes in Chinese Young Adults: Cross-Sectional Study
Source: JMIR Public Health Surveill. 2024 Jan 26;10:e50020. doi: 10.2196/50020 (PMC10858424; doi:10.2196/50020)
Supplement: Multimedia Appendix 4 [file publichealth_v10i1e50020_app4.docx]

| **Multimedia Appendix 4.** Latent class prevalence of the 3-class model without measurement invariance across sex and the conditional item probabilities of online sexual experiences in female participants. | | | |
| --- | --- | --- | --- |
| Item probabilities of online sexual experiences (yes %): | Class 1: Abstinent  (N = 274) | Class 2: Normative  (N = 284) | Class 3: Active  (N = 65) |
| Latent class prevalence: | 44.0% | 45.5% | 10.5% |
| 2. Exposed to pornographic content online or on social media | 4.3% | **98.1%** | **96.3%** |
| 6. Accessed sexuality content online or on social media | 3.3% | **85.2%** | **94.4%** |
| 1. Discussed sex with others on social media | 10.0% | **60.6%** | **86.5%** |
| 9. Actively sought pornographic content online or on social media | 2.7% | 39.3% | **86.7%** |
| 3. Dated people acquainted with online or on social media | 7.6% | 29.7% | **75.9%** |
| 11. Exposed to pornographic content in internet games | 3.2% | 25.7% | **68.5%** |
| 4. Received pornographic (text or video) messages online | 1.9% | 15.9% | **55.2%** |
| 10. Posted/shared indecent photos online or on social media | 0.3% | 11.0% | **60.4%** |
| 7. Had sex with people acquainted with online or on social media | 0.4% | 6.8% | 35.1% |
| 5. Sent pornographic (text or video) messages online | 0.3% | 3.2% | 33.9% |
| 8. Had naked chat online | 0.3% | 0.0% | 25.1% |
| N = 623; Substantial conditional item probabilities that are greater than .40 are bolded for the latent classes. Items were presented in descending order for the crude unweighted probabilities. | | | |
